# A deep convolutional visual encoding model of neuronal responses in the LGN

**DOI:** 10.1186/s40708-021-00132-6

**Published:** 2021-06-15

**Authors:** Eslam Mounier, Bassem Abdullah, Hani Mahdi, Seif Eldawlatly

**Affiliations:** 1 Computer and Systems Engineering Department, Faculty of Engineering, Ain Shams University, 1 El-Sarayat St., Abbassia, Cairo, Egypt; 2 Faculty of Media Engineering and Technology, German University in Cairo, Cairo, Egypt

**Keywords:** Convolutional neural network, Encoding, LGN, Modeling, Spike trains

## Abstract

The Lateral Geniculate Nucleus (LGN) represents one of the major processing sites along the visual pathway. Despite its crucial role in processing visual information and its utility as one target for recently developed visual prostheses, it is much less studied compared to the retina and the visual cortex. In this paper, we introduce a deep learning encoder to predict LGN neuronal firing in response to different visual stimulation patterns. The encoder comprises a deep Convolutional Neural Network (CNN) that incorporates visual stimulus spatiotemporal representation in addition to LGN neuronal firing history to predict the response of LGN neurons. Extracellular activity was recorded in vivo using multi-electrode arrays from single units in the LGN in 12 anesthetized rats with a total neuronal population of 150 units. Neural activity was recorded in response to single-pixel, checkerboard and geometrical shapes visual stimulation patterns. Extracted firing rates and the corresponding stimulation patterns were used to train the model. The performance of the model was assessed using different testing data sets and different firing rate windows. An overall mean correlation coefficient between the actual and the predicted firing rates of 0.57 and 0.7 was achieved for the 10 ms and the 50 ms firing rate windows, respectively. Results demonstrate that the model is robust to variability in the spatiotemporal properties of the recorded neurons outperforming other examined models including the state-of-the-art Generalized Linear Model (GLM). The results indicate the potential of deep convolutional neural networks as viable models of LGN firing.

**Supplementary Information:**

The online version contains supplementary material available at 10.1186/s40708-021-00132-6.

## Introduction

Modeling the encoding of the external visual stimulus along the visual pathway has been the topic of a multitude of studies given that humans arguably rely on vision more than any other sense [1–3]. With the recent advances in neural interfaces technology and the development of visual prostheses to restore vision to the blind [4, 5], more attention has been given to the development of accurate visual encoding models that could predict the activity of neurons to arbitrary visual inputs along the visual pathway. For instance, including an encoding stage prior to retinal stimulation to identify the expected firing pattern of retinal ganglion cells (RGCs) has been demonstrated to result in RGCs activity that closely mimics natural responses [6, 7]. In addition, it has been demonstrated that modeling the encoding of RGCs firing could be used to better tune inputs to cortical visual prostheses [8]. Finally, in thalamic visual prostheses, we have previously demonstrated that using Kalman filter-based and autoencoder-based encoders to tune electrical stimulation parameters could drive thalamic Lateral Geniculate Nucleus (LGN) neurons in a way similar to how natural visual inputs drive their activity [9, 10]. These findings across different visual prostheses types emphasize the need for encoding models of visual neurons as essential components of the design of visual prostheses.

The LGN has been demonstrated to be one of the major visual pathway sites used as a target for implanted visual prostheses [9, 11, 12]. However, it is much less studied compared to other visual pathway sites despite its crucial role as the major gateway of visual information to higher processing levels along the visual pathway [13]. It integrates and transforms the visual information flowing from the retina and passes such information to the primary visual cortex (V1) for further processing. Despite the simplistic role LGN neurons are argued to play, multiple studies demonstrated a rather complex role of the LGN in encoding visual information. Anatomically, it has been demonstrated that the LGN receives inputs from multiple brain areas such as the corticogeniculate feedback received from V1, which outnumbers retinal projections, and inputs from the brainstem, which modulate LGN activity independent of retinal input [14, 15]. In terms of firing patterns, LGN neurons have been demonstrated to exhibit both transient and sustained responses post-visual stimulus presentation [16, 17]. These findings suggest a more elaborate function of the LGN as opposed to acting as simple relay cells.

Extensive research in the past few decades has focused on developing models of neuronal firing in the visual pathway based on simultaneously recorded neuronal activity. Integrate-and-fire models have been demonstrated to predict neuronal responses corresponding to visual stimuli in multiple visual pathway sites [18, 19]. Statistical models have also been utilized to model the encoding of visual stimuli in neuronal spike trains. Generalized Linear Model (GLM) and its variants have been utilized in multiple studies to model neuronal firing along different sites of the visual pathway [1, 3]. It has been considered for years as the gold-standard statistical model for representing neural data [20, 21].

Recently, advances in machine learning techniques have motivated their use in modeling neuronal firing in multiple brain areas [22–24]. Convolutional Neural Networks (CNNs) in particular have demonstrated robust results in different domains including brain encoding [25, 26]. Compared to other statistical models of brain encoding, CNNs assume no prior knowledge about the distribution of the training data [24]. In addition, in studying visual encoding, inputs to visual encoding models typically represent images presented across time. Therefore, using CNNs would be the most suitable, since CNNs are by nature specialized to deal with spatiotemporal data that has grid-like topology. Moreover, CNNs could represent non-linear as well as linear relationships through the use of successive layers of units with non-linear activations [24, 25]. This is critical in modeling LGN neuronal firing given the growing evidence that using non-linear models can better characterize LGN firing compared to simple linear models [3, 27, 28].

In this paper, we introduce a deep CNN approach to represent spatiotemporal visual encoding in the LGN. We simultaneously recorded the extracellular activity of rat LGN neurons in response to single-pixel, checkerboard and geometrical shapes visual stimulation patterns. Data recorded using multi-electrode arrays from 12 anesthetized rats were pre-processed to extract instantaneous single-trial neuronal firing rates of the underlying population. Firing rates and the firing rate history, in addition to the corresponding visual stimulus spatiotemporal representation are then used to train the model. Our results indicate the efficacy of the proposed CNN visual encoder in predicting the neural firing activity of LGN neurons corresponding to single-trial visual stimulation patterns.

## Methods

### Rat lateral geniculate nucleus (LGN) in vivo recording

In this study, 12 adult female Albino rats (S1 to S12) were used with an average weight of ~ 100gm. All the experimental procedures were approved by the Research Ethics Committee at the Faculty of Medicine, Ain Shams University, Cairo, Egypt (Approved on February 15, 2017). Animals were anesthetized using Urethane (1 g/kg injected intraperitoneally and supplemented as needed). The animal was fitted in a stereotaxic instrument (Digital Lab Standard Stereotaxic, Stoelting Co, Wood Dale, IL, USA) and its body temperature was kept at ~ 37 ˚C. An incision was made into the scalp and the underlying connective tissue was cut. A craniotomy of size 4 mm × 4 mm was then drilled over the right LGN (2–6 mm posterior and 1–5 mm lateral to bregma). The measured bregma–lambda distance was used as a scaling factor to map the coordinates of the LGN in the Rat brain atlas of Paxinos and Watson [29].

After resecting the dura to expose the brain surface, a 32-channel microelectrode silicon array (NeuroNexus Technologies, Ann Arbor, MI, USA) with four shanks, eight recording sites/shank, 200 μm shank separation and 50 μm within shank electrode separation was advanced into the right LGN in 100 μm/min steps. The ground wire of the electrode was connected to a screw fixed to the rat’s skull for noise elimination. The rat’s left eye was kept open during the recording using a hemostat attached to the skin. Signals acquired from the electrodes were amplified and bandpass filtered in the range 300–5000 Hz and sampled at 25 kHz (Tucker-Davis Technologies, Alachua, FL, USA).

LGN coordinates were identified at ~ 3.2 mm posterior and ~ 2.85 mm lateral to bregma at a depth of ~ 4 mm [29]. To identify the desired depth, a flashing light stimulus was applied, and the corresponding activity was observed. After reaching the desired depth, the visual stimulation patterns were presented using a 13-inch screen placed tangent to the rat’s left eye at ~ 15 cm distance (spanning ~ 88° of the visual field) in a completely dark room. At the end of the experiment, the rat was euthanized using an overdose intraperitoneal Sodium Thiopental injection.

### Visual stimulation patterns

We examined three types of visual stimulation patterns. The first type comprises single-pixel patterns, in which the screen is divided into 4 × 8 grid of pixels, where one pixel at a time is turned ON (white) for 200 ms and OFF (black) for 300 ms. The flickering of the same pixel is repeated for 100 times (trials) consecutively, followed by the flickering of the following pixel from the top left pixel on the screen till the bottom right pixel (see Additional file 1: Figure S1a). The single-pixel stimulation was applied to rats (S1 to S6). The second type is represented by checkerboard patterns of size 4 × 8 pixels. In each pattern, four pixels are randomly chosen to flicker simultaneously with durations of 200 ms ON and 300 ms OFF. A sequence of 32 different checkerboard patterns is presented consecutively. Patterns were generated such that all pixels within the whole sequence of patterns are guaranteed to flicker exactly the same number of times. The same checkerboard sequence is repeated for 100 times (trials) (see Additional file 1: Figure S1b). The checkerboard stimulation was applied to rats (S7 to S12). A third type of stimulation is represented by arbitrary geometrical shapes (rectangle, circle, triangle and cross) in whole screen resolution. For each stimulation pattern, a random shape is chosen to flicker with durations of 200 ms ON and 300 ms OFF in a random screen location (top–left, top–right, bottom–left, bottom–right or center). A sequence of 20 different shape patterns is presented consecutively (see Additional file 1: Figure S1c). The same sequence of shapes is repeated for 50 trials. The shapes stimulation was applied to rats (S7, S8 and S9).

The visual stimulation types examined are consistent with the patterns utilized in previous studies [30–32]. Stimulation patterns were preceded by full-field stimulation. In this pattern, the whole screen is turned ON for 200 ms followed by OFF for 300 ms. The full field pattern is repeated 80 times. This full-field stimulation is used to define the responsive neurons and to synchronize the recorded activity with the visual stimulus.

### Data pre-processing

The raw stimulus-driven activity recorded during the experiment was then passed through multistage pre-processing. This is to convert the recorded signals to spike trains. A spike train is a binary sequence of instances at which a neuron is detected to fire an action potential, where ‘1’ indicates a spike, while ‘0’ indicates no spike. We used NeuroQuest, which is a MATLAB toolbox for neural data processing and analysis for this task [33, 34]. First, spikes were extracted from each recording site filtered signal. A spike was detected if the signal surpasses a tunable threshold set at three times the signal standard deviation of the noise. Spikes were extracted at an interval starting at 0.75 ms pre-threshold to 2 ms post-threshold crossing. Spikes detected from each site were then aligned at their trough and Principal Component Analysis (PCA) was applied to the extracted aligned spikes. We used the first two principal components as features for K-means clustering algorithm to identify different clusters of spikes within the recording site [35]. Each cluster of spikes was considered as the spiking of a single unit (neuron) recorded on this site. Number of single units recorded on each recording site was manually determined. Spike trains were then extracted with a resolution of 1 ms. Finally, we computed the firing rate of each neuron from the corresponding spike train. The firing rate at any time bin *k* is the spike count of this neuron in the interval ((*k* *–* 1) × *w*) – (*k* × *w*) divided by *w*, where *w* is the firing rate bin width. In our analysis, we examined two different values of *w*; 10 ms (fine resolution) and 50 ms (coarse resolution) [36, 37].

### Responsive neurons spatiotemporal properties

Spike raster and Post-stimulus Time Histogram (PSTH) analyses of the full-field stimulation were used to select neurons with a clear stimulus-driven response from the total population [38]. PSTH and spike rasters were also used for the alignment of the spike trains with the onset of the visual stimulus.

Further responsiveness analysis was carried out that is based on the periodic nature of the presented stimulus. Since the stimulus is repeated every 500 ms, it is expected that responsive neurons would exhibit spiking responses that have a similar periodic nature. The periodicity of firing is assessed using Power Spectral Density (PSD) [39]. A responsiveness-index *R* was computed in decibels from the PSD of the firing rate of neurons based on the dominant frequency components:

1 $$R = 10\log_{10} \max \left( {{\text{PSD}}\left( f \right) , {\text{PSD}}\left( {2f} \right) , {\text{PSD}}\left( {3f} \right)} \right)$$

where *f* is the frequency of the periodic visual stimulus (i.e., 2 Hz). The frequencies 2*f* and 3*f* are considered in this analysis as they represent the harmonics of the stimulus frequency. Responsive neurons are expected to have high *R* due to high power at the frequencies corresponding to the visual stimulus.

The spatiotemporal properties of the recorded neurons were also examined. Neurons were classified into two groups based on their temporal properties: transient neurons in which the spiking increases after the onset/offset of visual stimulus, and sustained neurons in which the spiking is relatively maintained during the stimulus duration. This was quantified using a transient-sustained ratio computed from the spike train of each neuron from the full-field stimulation trials as

2 $${\text{tsr}} = \frac{{{\text{Spk}}_{t} }}{{{\text{Spk}}_{s} }}$$

where tsr is the transient-sustained ratio, ${\text{Spk}}_{t}$ is the average number of spikes across trials for a specific neuron computed within a transient window = 100 ms after the stimulus (onset/offset), and ${\text{Spk}}_{s}$ is the corresponding average number of spikes across trials during the entire stimulus duration (ON/OFF). A neuron was classified as transient if the transient-sustained ratio > 0.5; otherwise, it was classified as sustained.

Spatial properties of the recorded neurons were also examined. We used the spike-triggered average (STA) to interpret each neuron’s visual receptive field [40]. STA of each neuron for each pixel is simply computed by extracting stimulus signal within the trial duration preceding each spike and then averaging the extracted signals across all spikes. By integrating the STAs over time for each neuron, we obtained the normalized receptive field matrix. To classify a neuron as ON or OFF neuron, we examined the STA of this neuron with the pixel that has maximum response. High STA value at the onset of the stimulus indicates an ON neuron, while low STA value at the onset of the stimulus indicates an OFF Neuron.

### Convolutional neural network visual encoder model

**Fig. 1:**
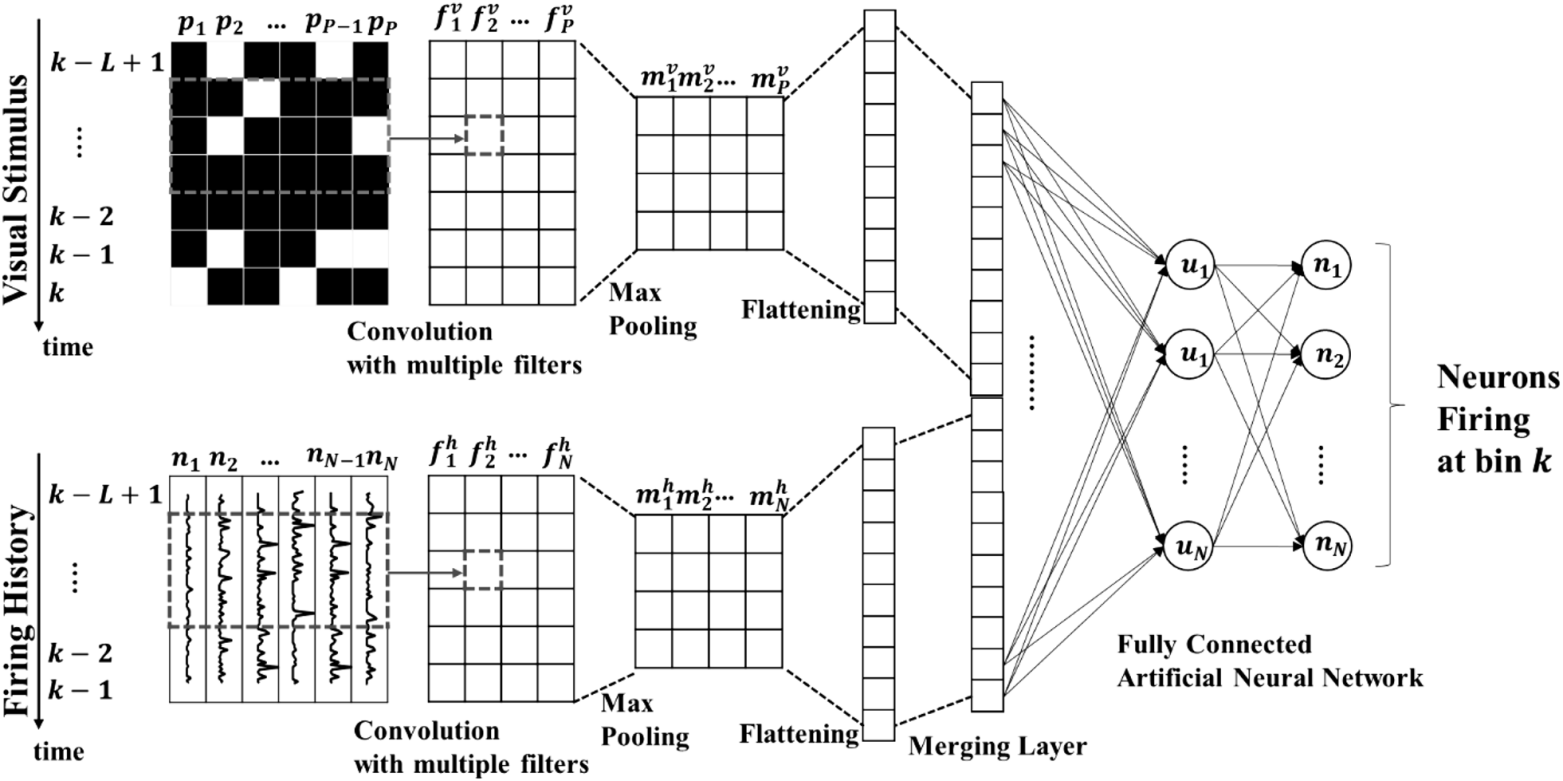
Topology of the deep convolutional visual encoding model. The visual stimulus input matrix passes through a 1D convolution process, followed by a maximum pooling stage then a flattening stage to produce a 1D visual feature vector. The firing history input matrix passes through similar stages to produce a 1D firing feature vector. The two feature vectors are concatenated to produce a combined feature vector which is presented to a fully connected neural network to finally predict firing rate values for all neurons

The purpose of the proposed visual encoder is to predict the firing rate of each recorded LGN neuron at time bin *k* for each individual subject based on the presented visual stimulus spatiotemporal representation as well as the firing history of the neurons. In the introduced encoder, we employed a deep convolutional neural network (CNN) architecture. We refer to this model as CNN-visual stimulus-firing history, denoted hereafter by CNN-V-FH. The model topology is illustrated in Fig. 1. In all layers, we used the Parametric Rectified Linear Unit (*PReLU*) activation *f* which takes the form [41]:

3 $$f\left( x \right) = {\text{PReLU}}\left( x \right) = \left\{ {\begin{array}{*{20}c} {x, \,x > 0} \\ {\alpha x, \,x \le 0} \\ \end{array} } \right.$$

where *x* is the input of *f* and *α* is a learnable parameter controlling the slope of the negative part of the function. The use of PReLU was introduced to solve the disadvantage of the popular traditional ReLU activation of producing zero output for all negative input values. This problem is called “dying ReLU”. For instance, the usage of PReLU outperformed the original ReLU with nearly zero extra computational cost producing better error rates for ImageNet 2012 with convolutional neural network models [41].

A model was trained for each individual subject using single trials, where two types of inputs were presented to the model at any given bin *k* in time: the first input is the Visual Stimulus matrix, which represents the visual stimulus spatiotemporal pattern presented to the rat. This matrix contains the intensity of each pixel of the stimulus during the interval $k$ to $k - L + 1$, where *L* is the total number of time bins equivalent to 500 ms (10 bins in case of *w* = 50 ms and 50 bins in case of *w =* 10 ms). The size of the Visual Stimulus matrix is thus *L* × *P*, where* P *is the number of pixels in the 4 × 8 visual stimulation grid (32 pixels). The second input to the model is the Firing History matrix, which contains the firing rate of all neurons in the interval* k *− 1 to* k *− *L* +1. The size of the matrix is (*L* − 1) × *N*, where *N* is the number of neurons per subject.

A 1D temporal convolution operation is applied to the Visual Stimulus matrix, where the number of convolutional filters is set to be equal to the number of pixels *P* (i.e., 32 filters). The width of the temporal convolutional filters is constant equal to the width of the Visual Stimulus matrix, while the length of the filters is varied from 3 to 8 which is along the direction of the 1D convolution (i.e., time axis). The result of this stage is a matrix of size *L* × *P*. In this matrix, the values in column $i$ represent the result of convolving filter $f_{i}^{v}$ with the Visual Stimulus matrix. The PReLU activation was then applied to the resulting matrix. A maximum pooling operation is then performed to compute the maximum value in the output of the preceding layer with a pooling size of 2 and a stride value of 2 to avoid overlapping. The size of the matrix resulting from this operation is (*L/*2) × *P*. The combination of convolution followed by maximum pooling is used once in the case of *w* = 50 ms and cascaded three times in the case of *w* = 10 ms. When *w* = 50 ms, the length of the resulting matrix after maximum pooling is 5 × *P*, since the input matrix in this case has 10 bins (500 ms divided by *w*). Thus, applying another layer of convolution-maximum pooling would result in diminished data length with no significant feature representation. However, in the case of *w* = 10 ms, the resulting matrix after the first convolution-maximum pooling layers would result in a matrix of size 25 × *P﻿,* since in that case, the input matrix has 50 bins. Thus, applying additional convolution-maximum pooling layers could capture higher level features in such finer resolution case.

The output of the final maximum pooling stage is then flattened into a 1D vector of visual stimulus features. This same process is applied to the Firing History Matrix with similar architecture, where the number of convolutional filters is equal to the number of neurons *N*. The final feature vector is then obtained by concatenating the visual stimulus and firing history flattened feature vectors. A 2-layer fully connected neural network is applied to the final feature vector, where both the hidden and output layers consist of *N* PReLU units. Each of the output layer units represents the predicted firing of one neuron at time bin *k*. To summarize, the model consisted of a 1D CNN layer, followed by a maximum pooling layer, followed by a flattening layer for the visual and firing history branches. A concatenation layer is applied to the output of both branches. A 2-layer fully connected network is then applied to produce the firings rates. The total number of layers is 9 in the case of *w* = 50 ms. While in the *w* = 10 ms case, the cascading of the CNN—maximum pooling blocks three times for the visual and firing history branches increases the total number of layers to 17.

The model was implemented in Python using Keras [42], and trained to optimize all the weights and the biases using Adam stochastic optimizer to minimize the root-mean-square error loss function [43]. The learning rate of the optimizer was set to 0.001 and the exponential decay rate for the first and second moment estimates was set to 0.9 and 0.999, respectively. In all layers, Xavier uniform weights initialization was used, with all biases initialized to zero.

It should be noted that we examined the use of batch normalization and dropout techniques to enhance the model performance. However, no significant enhancement was observed. We also examined different filter lengths as indicated previously (3–8) in the 1D convolution process, but we included hereafter the results of the best filter lengths. For comparison, we also examined variants of the CNN-V-FH model that use the same topology of Fig. 1 but with the Visual Stimulus Matrix (CNN-V) only as an input, and another variant with the Firing History Matrix (CNN-FH) only as an input.

### Poisson generalized linear visual encoder model

The proposed deep learning model was compared to the Generalized Linear Model (GLM) that has been used for modelling neuronal firing in numerous studies [1, 3]. In this approach, the statistical learning model of the GLM is used to fit and predict the neuronal firing corresponding to the presented visual stimulus and the neuronal firing history. In this model, the firing rate of any neuron *i* at any timepoint *t* is expressed using a conditional mean intensity function:

4 $$\lambda_{i} \left( {t|V_{i} \left( t \right),H_{i} \left( t \right)} \right) = {\text{exp}}\left( {\beta_{i} + V_{i} \left( t \right) + H_{i} \left( t \right)} \right)$$

where $\beta_{i}$ is the background firing rate of neuron *i*, $V_{i} \left( t \right)$ denotes the presented visual stimulation pattern parameters, and $H_{i} \left( t \right)$ denotes the firing history of all neurons including neuron *i:*

5 $$H_{i} \left( t \right) = \mathop \sum \limits_{n = 1}^{N} \mathop \sum \limits_{m = 1}^{L} \beta_{in}^{h} \left( {mw} \right) S_{n} \left( {t - mw} \right)$$

6 $$V_{i} \left( t \right) = \mathop \sum \limits_{p = 1}^{P} \mathop \sum \limits_{m = 0}^{L} \beta_{ip}^{v} \left( {mw} \right) I_{p} \left( {t - mw} \right)$$

where $S_{n} \left( {t - mw} \right)$ is the firing of neuron *n* in history bin *m* and $\beta_{in}^{h}$ is the weight of the contribution of this neuron firing to the overall function $H_{i} \left( t \right)$. Similarly, $I_{p} \left( {t - mw} \right)$ is the intensity of pixel *p* in history bin *m* and $\beta_{ip}^{v}$ is the weight of the contribution of this pixel intensity to the overall function $V_{i} \left( t \right)$ (see Additional file 1 for details of GLM training).

### Performance assessment of the visual encoder models

We examined the performance of the models using first the single pixel and checkerboard data sets using 5-fold cross-validation with non-overlapping training/test sets. In single-pixel experiments, we used 80% of the trials of each pixel for training in each fold and kept the remaining 20% for testing. In the case of checkerboard experiments, we used all trials of 80% of the patterns in each fold for training and we kept all trials of the remaining 20% of the patterns for testing. For all encoding models, we used the same training and testing data sets.

The process of model assessment was performed as follows: every sample at time bin *k* in the testing portion of the data set consisting of the visual stimulus spatiotemporal representation and the firing history of all neurons was presented to the model to generate a prediction of the firing rate to be compared with the corresponding actual firing rate. This process is repeated for all data samples for all neurons in each fold of the cross-validation analysis. The correlation coefficient between the actual and predicted firing rates averaged across all test trials and all fivefolds of cross-validation was used to evaluate the performance of the model [18, 44].

In our analysis of the recorded neurons performance based on their temporal characteristics, we aimed to eliminate the effect of the responsiveness of the neurons. This was done by scaling the obtained correlation coefficient based on the normalized value of its responsiveness index. The adjusted correlation can be expressed as

7 $${\text{adjusted}}_{{{\text{corr}}}} = \frac{{corr + \left( {1 - R_{{{\text{norm}}}} } \right)}}{2}$$

where $R_{{{\text{norm}}}}$ is the normalized responsiveness index computed from the responsiveness index *R* of Eq. (1) by subtracting the minimum responsiveness value and then dividing by maximum value. This is to ensure that the range of adjusted correlation measure ranges from 0 to 1, where the correlations achieved for all analyzed neurons are from 0 to 1.

To assess the ability of the model to generalize, we also examined the performance of the model when tested using geometrical shapes. In this case, we trained the model using the whole checkerboard data set and tested the model on the more complicated shapes data set. The training procedure of the model was the same as described before, but using 100% of the checkerboard trials instead. To test the model, we projected every shape stimulus pattern into the low dimensional space (4 × 8) that the model was trained on.

## Results

### LGN neurons response characteristics

We recorded from a total of 150 well-isolated single units (neurons) from the right LGN in 12 anesthetized rats. Data was recorded in response to three visual stimulation patterns: single-pixel stimulation (Subjects S1 to S6, *n* = 86 neurons), checkerboard stimulation (Subjects S7 to S12, *n* = 64 neurons) and geometrical shapes stimulation (Subjects S7, S8 and S9, *n* = 29 neurons that are subset of the 64 neurons of checkerboard stimulation). An average neuronal population of size 12.5 ± 2.64 neurons per rat was extracted. Initial responsiveness of the recorded neurons to visual stimulation was assessed based on the analysis of the rasters and PSTHs extracted from spiking data of each neuron in response to initial full-field stimulation.

**Fig. 2:**
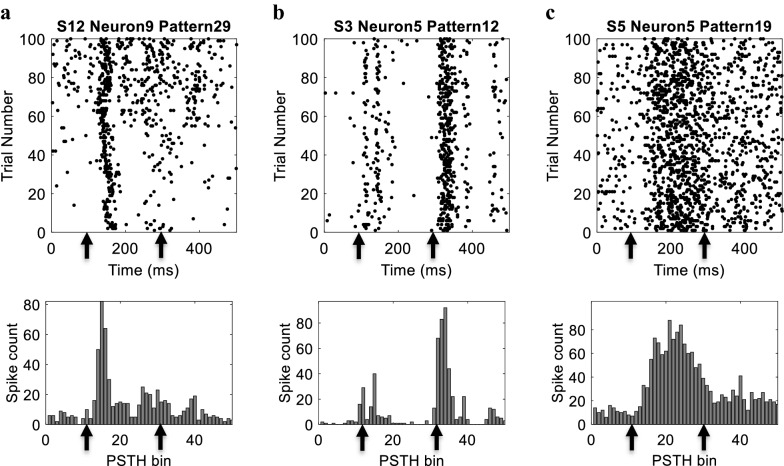
Responses of three sample neurons. (Top) Spike raster plots for multiple trials in response to specific stimulation pattern, where each dot represents a spike. (Bottom) Post-Stimulus Time Histograms (PSTH) with 10 ms bin width. **a** Transient neuron that responds more with stimulus onset (Neuron 9 in S12). **b** Sample transient neuron that responds more to the stimulus offset (Neuron 5 in S3). **c** Response of a sample sustained neuron (Neuron 5 in S5)

We first examined the temporal firing characteristics of the recorded neurons. Figure 2 shows the raster and PSTH plots of three sample neurons from different subjects. The raster plots show the spiking corresponding to a certain stimulus pattern across 100 different trials with a duration of 500 ms. The onset and offset times of the stimulus are marked with vertical arrows at 100 ms and 300 ms, respectively. The figure demonstrates the variability in the temporal firing characteristics where two different types of neurons were identified; namely, transient and sustained. Fig. 2a shows the firing of a transient response neuron in which a significant response is only observed post stimulus onset, while Fig. 2b shows the firing of another transient response neuron in which a significant response is only observed post stimulus offset. Finally, Fig. 2c shows the response of a sustained firing neuron which maintains its response for the duration of stimulus.

To classify neurons to one of the two types (transient versus sustained), we computed the transient-sustained ratio as the average number of spikes across trials fired after stimulus onset/offset to the average number of spikes fired across trials. The majority of neurons were classified as transient with a total of 102 neurons (68%) across all rats, while only 48 neurons (32%) were classified as sustained. We next examined the spatial firing characteristics of the recorded neurons in terms of their receptive fields. Receptive fields were reconstructed from the integration of the obtained STAs over time for each neuron. Based on the STAs, 87 neurons (58%) were classified as spatial-ON neurons, while 63 neurons (42%) were classified as spatial-OFF neurons. Additional file 1: Figure S2 shows the normalized receptive fields of the neurons shown in Fig. 2 and the normalized receptive field of all neurons across all rats.

### Performance of the CNN model

A deep convolutional visual encoding model was trained using the recorded data. For each visual stimulation pattern, 100 trials of duration 500 ms were extracted from single pixel and checkerboard stimulation. Firing rates were then computed with bins of width *w* = 50 ms and *w* = 10 ms. For each rat, two models were trained following the architecture shown in Fig. 1 (CNN-V-FH); one for each value of *w*. Fivefold cross-validation was adopted to train the model using 80% of the data. The visual stimulation patterns of the remaining 20% were then used as input to the model to predict the firing of the recorded neurons.

**Fig. 3:**
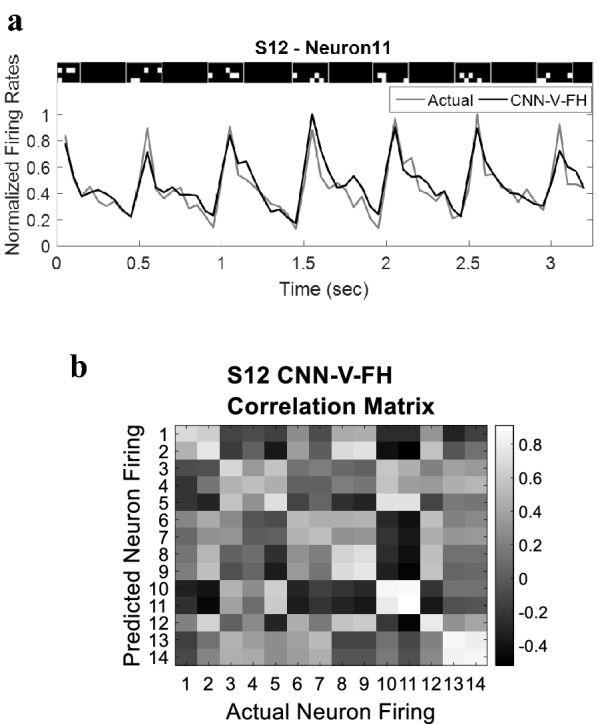
Firing rate prediction. **a** Predicted firing rate (black) versus the actual firing rate (gray) corresponding to the shown checkerboard stimulation patterns in subject S12, Neuron 11, with firing rate window *w* = 50 ms. Each stimulation pattern remains ON for 200 ms followed by 300 ms of no stimulus (OFF). **b** Correlation matrix of subject S12 obtained using the CNN-V-FH visual encoder model

To evaluate the performance of the proposed model, we computed the correlation coefficient between the predicted firing and the actual recorded firing of each neuron averaged across all test trials and cross-validation folds. Figure 3a demonstrates the normalized actual firing rate and the firing rate predicted using the model for the best performing neuron in subject S12. The figure shows significant similarity between the actual and predicted firing rates.

The performance of the model was assessed at the population level by computing the correlation matrix for the data set of each rat in which each value represents the correlation between actual and predicted firing rates of the corresponding pair of neurons. Figure 3b shows the correlation matrix obtained in rat S12 for the 14 neurons recorded with *w* = 50 ms. The diagonal-like form of the correlation matrix indicates the ability of the model to learn the intrinsic firing properties of each neuron rather than learning the underlying common mode firing of the population.

**Fig. 4:**
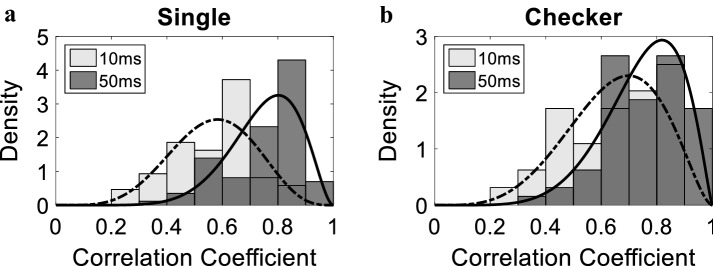
Histogram of the achieved correlation coefficients using CNN-V-FH for all neurons and the fitted beta distribution. Solid and dotted curves represent fits of the 50 ms and 10 ms histograms, respectively. **a** Single-pixel stimulation (*α* = 5.69 and *β* = 4.33 for *w* = 10 ms, and *α* = 7.45 and *β* = 2.48 for *w* = 50 ms). **b** Checkerboard stimulation (*α* = 4.46 and *β* = 2.41 for *w* = 10 ms, and *α* = 6.33 and *β* = 2.13 for *w* = 50 ms)

In our analysis, two different resolutions for the firing rate window were used: *w* = 50 ms and *w* = 10 ms. Figure 4 shows a histogram of the correlations between predicted and actual firing rates of all neurons in all subjects. The figure shows the histograms for both single pixel (Fig. 4a) and checkerboard (Fig. 4b) visual stimulation patterns for each firing rate window. The figure demonstrates significant similarity between predicted and actual firing rates as indicated by the fitted Beta distributions. Prediction correlation reached a maximum of 0.945 in the *w* = 50 ms case and 0.884 in the *w* = 10 ms case. For both single pixel and checkerboard stimulation patterns, a better performance is observed for *w* = 50 ms (single-pixel correlation: 0.75 ± 0.13, checkerboard correlation: 0.75 ± 0.14) compared to *w* = 10 ms (single-pixel correlation: 0.57 ± 0.15, checkerboard correlation: 0.65 ± 0.17). This is expected given the noise reduction when computing the firing rate in a 50 ms window compared to a narrower 10 ms window.

**Fig. 5:**
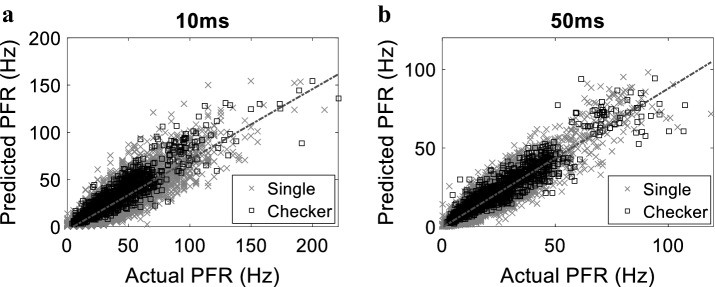
Predicted versus the Actual Peak Firing Rates (PFRs) following the stimulus for **a** *w* = 10 ms and **b** *w* = 50 ms. Each point in **a** and **b** corresponds to one neuron peak spiking for a specific stimulation pattern. Dotted lines indicate linear regression lines

To evaluate the performance of the model furthermore, we compared the actual and predicted peak firing rate of every neuron post the onset of each stimulation pattern. Figure 5 demonstrates that the model predicts the peak firing rate with a significant similarity (*w* = 10 ms: *r *^2^ = 0.78, *P* < 1e^−33^ *w* = 50 ms: *r *^2^ = 0.88, *P* < 1e^−33^). In addition, we assessed the ability of the model to capture the trial-to-trial variability across stimulation patterns. Significant similarity between the trial-to-trial variability in predicted firing and that in actual firing was detected (*w* = 10 ms: *r* ^2^ = 0.6, *P* < 1e^−33^; *w* = 50 ms: *r *^2^ = 0.67, *P* < 1e^−33^) (See Additional file 1: Figure S3).

### Relating model performance to neuronal responsiveness

**Fig. 6:**
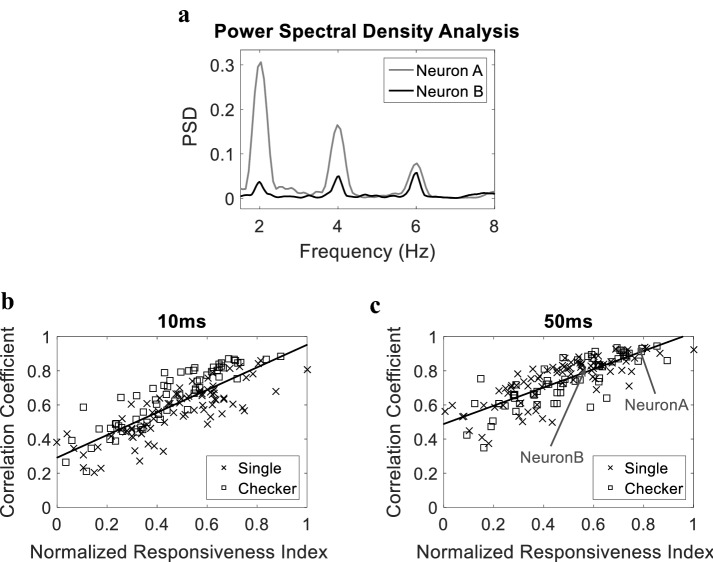
Performance as a function of responsiveness. **a** Power spectral density calculated from the firing rate of 2 neurons; neuron A (gray) and neuron B (black). **b** Achieved correlation coefficients using CNN-V-FH for all neurons versus normalized responsiveness index for all neurons with firing rate windows *w* = 10 ms and **c** *w* = 50 ms. Each point in **b** and **c** corresponds to one neuron. Solid lines indicate linear regression lines. Arrows in **c** point to the neurons whose PSD is shown in **a**

The responsiveness of the recorded neurons to stimulation patterns was not uniform across all populations. Thus, given the periodic nature of the stimulus applied, we first quantified the responsiveness of the neurons using a responsiveness index based on the power spectral density (PSD) of the spike train of each neuron. We next examined the impact of the responsiveness of the neurons on the performance of the model. Figure 6a shows the PSD of two neurons calculated from their corresponding firing rates with *w* = 50 ms. The figure demonstrates a higher power for neuron A compared to neuron B at 2 Hz (the stimulus frequency). This indicates that neuron A is more responsive to the presented stimuli compared to neuron B. A relatively high responsiveness index is obtained for neuron A of 0.306 ( − 5.23 dB) compared to 0.058 ( − 12.36 dB) for neuron B. Figure 6 shows how the model performance measured as the correlation between predicted and actual firing rates varies with the neuronal normalized responsiveness index. The figure demonstrates that the model predicts the firing of highly responsive neurons with higher accuracy compared to less-responsive neurons in both cases *w* = 10 ms, as shown in Fig. 6b (*r *^2^ = 0.6503, *P* < 1e^−33^), and *w* = 50 ms, as shown in Fig. 6c (*r *^2^ = 0.6512, *P* < 1e^−33^). This was observed for both single pixel and checkerboard visual stimulation patterns. These results indicate that the low correlations obtained for some neurons could be attributed to their diminished response to visual stimulation rather than improper training of the model.

### Relating model performance to spatiotemporal firing characteristics

**Fig. 7:**
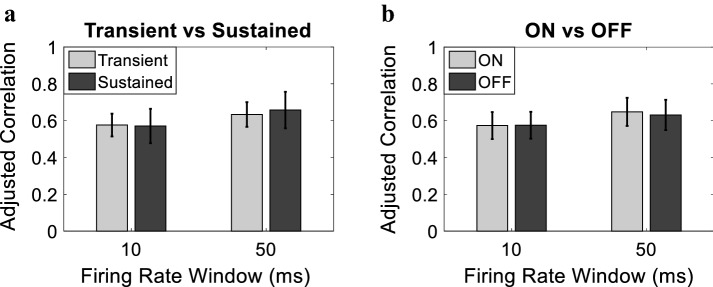
Model performance expressed as the adjusted correlation coefficient scaled by responsiveness index for different spatiotemporal firing characteristics. **a** Transient and sustained neurons for both *w* = 10 ms and *w* = 50 ms (mean ± SD). **b** ON and OFF neurons achieved for both *w* = 10 ms and *w* = 50 ms (mean ± SD)

Given the variability in the spatiotemporal firing characteristics of the recorded neurons, we examined the corresponding effect on the model performance. We first assessed the performance as a function of the temporal characteristics, as shown in Fig. 7a, to determine if the model is biased towards one of the two types (transient or sustained). Given that the majority of the sustained firing neurons have diminished responsiveness index compared to their transient counterpart, we sought to eliminate the effect of the responsiveness when assessing the model performance. We used an adjusted correlation coefficient measure in which we scaled the correlation coefficient between the predicted and actual firing rates of a given neuron by its corresponding responsiveness index. Figure 7a demonstrates the firing rate prediction of the model for transient and sustained neurons. The figure illustrates no significant difference between adjusted correlations of the two types for both *w* = 10 ms (sustained: 0.58 ± 0.06, transient: 0.57 ± 0.09, *P* > 0.3) and *w* = 50 ms (sustained: 0.66 ± 0.1, transient: 0.63 ± 0.07, *P* > 0.1). In terms of spatial characteristics, Fig. 7b shows no significant difference between the performance of the model for ON and OFF neurons. This is observed for *w* = 10 ms (ON: 0.57 ± 0.07, OFF: 0.58 ± 0.07, *P* > 0.5) and *w* = 50 ms (ON: 0.65 ± 0.08, OFF: 0.63 ± 0.08, *P* > 0.05). These results indicate that the model is not biased to specific temporal or spatial firing characteristics.

### Comparison with other models

**Fig. 8:**
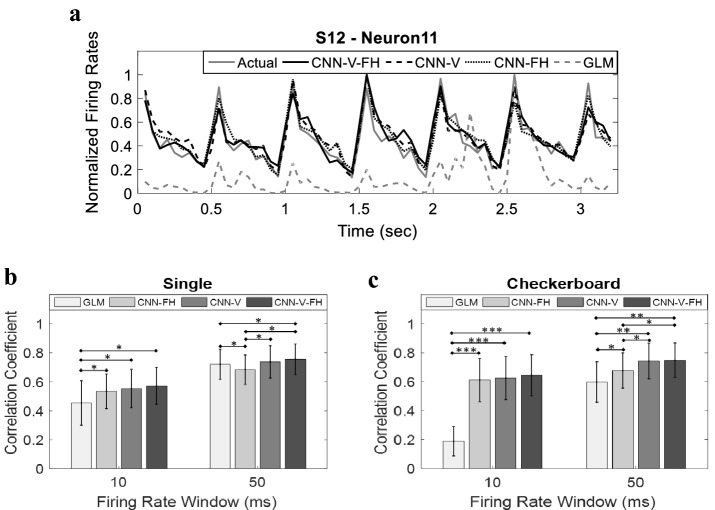
Performance of different models. **a** Firing rates predicted using the four models compared to the actual firing rate in a sample neuron (Neuron 11) in subject S12. Firing rates correspond to checkerboard visual stimulation pattern with a firing rate window of size *w* = 50 ms. (**b**) Correlation coefficient averaged across all neurons in the single-pixel stimulation pattern for both *w* = 10 ms and *w* = 50 ms (mean ± SD). *P < 0.05, Wilcoxon rank-sum test. (**b**) Correlation coefficient averaged across all neurons in the checkerboard stimulation pattern for both *w* = 10 ms and *w* = 50 ms (mean ± SD). **P < 1e^−5^, ***P < 1e^−19^, Wilcoxon rank-sum test

We compared the performance of the proposed CNN-V-FH model to three other models. All three models predict the firing of the entire population of each subject. The first is a deep CNN model similar to the architecture shown in Fig. 1, but without taking into account the spiking history of the modeled neurons. This model is referred to as CNN-V. The second is also a similar deep CNN model but without the visual stimulation; referred to as CNN-FH. The third model is the classical Generalized Linear Model (GLM) which has been employed in a multitude of studies to model neuronal firing in the visual pathway [1, 45]. The CNN-V, CNN-FH and GLM models were trained using the same training data set that was used to train the CNN-V-FH model. The models were then used to predict the firing rates of all neurons in the testing data sets. Figure 8a shows the actual and predicted firing rates estimated using the four models for a sample neuron (same neuron demonstrated in Fig. 3a with 0.91 correlation for CNN-V-FH. The figure demonstrates the similarity between the firing rates predicted using the deep CNN models, and the actual firing rate, as opposed to that predicted using the GLM.

Comparing the CNN-V-FH model accuracy to the other CNN variants, a slight but not significant improvement was observed over CNN-V, while a more significant improvement over CNN-FH was observed for both single-pixel stimulation, as shown in Fig. 8b (*w* = 10 ms—CNN-V-FH: 0.57 ± 0.13, CNN-V: 0.55 ± 0.13, CNN-FH: 0.53 ± 0.12; *w* = 50 ms—CNN-V-FH: 0.76 ± 0.11, CNN-V: 0.74 ± 0.11, CNN-FH: 0.68 ± 0.1) and checkerboard stimulation, as shown in Fig. 8c (*w* = 10 ms—CNN-V-FH: 0.65 ± 0.14, CNN-V: 0.63 ± 0.15, CNN-FH: 0.61 ± 0.15; *w* = 50 ms—CNN-V-FH: 0.75 ± 0.12, CNN-V: 0.74 ± 0.12, CNN-FH: 0.68 ± 0.12). On the other hand, a significant improvement was observed comparing the CNN-V-FH model to the GLM model in the single-pixel stimulation pattern at *w* = 10 ms and to less extent at *w* = 50 ms (*w* = 10 ms—CNN-V-FH: 0.57 ± 0.13, GLM: 0.45 ± 0.15, *P* < 0.001; *w* = 50 ms—CNN-V-FH: 0.76 ± 0.11, GLM: 0.72 ± 0.1). For the checkerboard stimulation pattern, significant improvement was observed using CNN-V-FH compared to GLM (*w* = 10 ms—CNN-V-FH: 0.65 ± 0.14, GLM: 0.19 ± 0.1, *P* < 1e^−19^; *w* = 50 ms—CNN-V-FH: 0.75 ± 0.12, GLM: 0.6 ± 0.14, *P* < 1e^−5^).

**Fig. 9:**
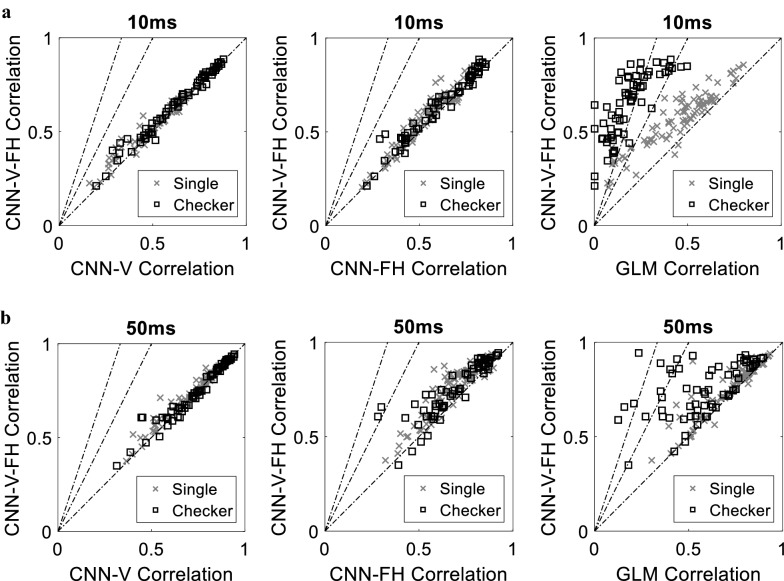
Prediction of all neurons in the CNN-V-FH model compared to the CNN-V, CNN-FH and GLM models for **a** *w* = 10 ms and **b** *w =* 50 ms. Each point in the plots represents one neuron. The three dotted lines with different slopes represent how much enhancement is achieved. The 45° line represents equal correlation between the corresponding pair of examined methods. The two other lines represent two and three times enhancement in the prediction correlation for the CNN-V-FH model compared to the other model

We next quantified the enhancement in the prediction correlation of the CNN-V-FH compared to the three other models at the single neuron level. Figure 9 illustrates the prediction correlation for each neuron comparing CNN-V-FH to the other methods. We observed a significant enhancement in the prediction correlation at the single neuron level in the CNN-V-FH compared to the GLM model reaching an enhancement in 100% of the recorded neurons with the checkerboard stimulation pattern for *w* = 10 ms. Moreover, despite the slight enhancement in the overall prediction correlation using CNN-V-FH compared to CNN-V and CNN-FH, the prediction correlation of a significant percentage of the recorded neurons was enhanced. The prediction correlation at *w* = 10 ms of 79.1% and 79.7% of the neurons was enhanced using the CNN-V-FH compared to the CNN-V model in the single pixel and checkerboard stimulation patterns, respectively, and of 86.1% and 78.1% of the neurons compared to the CNN-FH model in the single pixel and checkerboard stimulation patterns, respectively. For the *w* = 50 ms case, the prediction correlation of 81.4% and 54.7% of the neurons was enhanced using the CNN-V-FH compared to CNN-V in the single pixel and checkerboard stimulation patterns, respectively, and of 89.5% and 87.5% of the neurons compared to the CNN-FH model in the single pixel and checkerboard stimulation patterns, respectively. This indicates the significance of incorporating the firing history in addition to the input visual stimulus into the model.

### Assessment of the model generalization ability

**Fig. 10:**
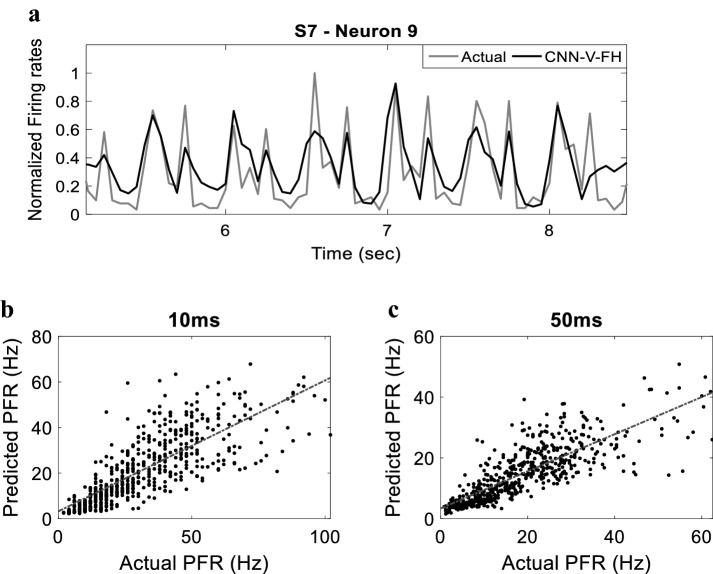
Model predictions to geometrical shapes stimulation. **a** Sample of the predicted firing rate (black) versus the actual firing rate (gray) corresponding to geometrical shapes stimulation patterns in subject S7, neuron 9, with firing rate window *w* = 50 ms. The predicted versus actual peak firing rates (PFRs) following the stimulus for **b** *w* = 10 ms and **c** *w* = 50 ms. Each point corresponds to one neuron peak firing for a specific shape pattern. Dotted lines indicate linear regression lines

The achieved results using the checkerboard stimulation patterns indicate the ability of the model to generalize given the lack of overlap between the training and testing patterns. To further confirm the generalization ability of the model, the CNN-V-FH model was examined by training the model using checkerboard stimulation patterns and testing it using the more complicated shapes patterns in three subjects (S7, S8 and S9). Figure 10a demonstrates the normalized actual and predicted firing rates corresponding to geometrical shapes stimulation patterns using the CNN-V-FH model for the best performing neuron in subject S7 with *w* = 50 ms. The figure shows significant similarity (0.65 correlation) between the actual and predicted firing. To evaluate the performance for the entire population, we computed the correlation coefficient between the actual and predicted firing rates corresponding to shapes stimulation for all neurons in the three subject’s prediction.

Correlation reached a maximum of 0.65 and 0.63 in the *w* = 50 ms and *w* = 10 ms cases, respectively. The overall correlation across all subjects was 0.42 ± 0.11 for *w* = 10 ms and 0.43 ± 0.09 for *w* = 50 ms. In addition, we computed the peak firing rate following stimulation onset for every neuron. Figure 10 demonstrates that the model predicts the peak firing rate with significant similarity (*w* = 10 ms: *r *^2^ = 0.62, *P* < 1e^−33^; *w* = 50 ms: *r *^2^ = 0.66, *P* < 1e^−33^). These results demonstrate the ability of the model to generalize when applied to unseen patterns.

## Discussion

Unraveling the details of visual information encoding in the visual system has been a long-standing problem in systems neuroscience. Here, we aimed to develop a visual encoding model of the LGN. Using single-trial extracellular activity recorded from rat LGN, we trained a CNN deep learning model to predict LGN firing rates in response to different visual stimulation patterns. The proposed model showed efficacy in predicting neural firing at different temporal resolutions. In particular, the proposed CNN model that uses the visual stimulus as well as the firing history of the population as inputs achieved a mean correlation (between the actual and predicted firing rates) of 0.7 measured across all 12 examined rats and three different stimulation patterns. Our study represents, to our knowledge, the first utilization of deep learning techniques to model visual encoding in the LGN. Deep learning, and CNNs in particular, were used in multiple visual system studies to model visual encoding in other species such as modeling the firing of retinal ganglion cells in salamanders and mice [22] and V1 responses in macaques [23, 26]. One novel aspect of the proposed model in comparison to the aforementioned deep learning studies is the use of temporal convolution of firing rates history as an additional input to the model, which enhanced the overall performance compared to relying solely on the visual input.

In this study, we compared the performance of the proposed encoding models to the well-known generalized linear model (GLM). We found that the proposed CNN models predicted firing rates with significantly higher accuracy compared to the GLM. Our models far outperformed the GLM in the checkerboard stimulation patterns for both examined firing rate windows and in the simple single-pixel patterns at the fine 10 ms firing rate resolution. This indicates that the proposed models scale better in case of more complicated stimulus inputs and in case of finer firing rate resolutions compared to the GLM. Our results are consistent with previous reports that compared deep learning techniques to linear–nonlinear models such as the GLM in modeling neural encoding [24, 46]. The proposed model is highly non-linear which is manifested by the use of non-linear activation functions. Compared to the results obtained using the GLM model, the higher accuracies achieved using the proposed model indicates the underlying non-linearity in LGN activity. This represents another evidence that the LGN plays a more complex role in the visual pathway than providing a simple linear combination of retinal inputs.

One challenge that generally complicates the use of CNNs is determining the optimal CNN structure for a given data set as there is no agreement on a methodology that is guaranteed to result in the optimal model [47, 48]. Different trials that rely on grid search techniques are typically performed to identify the best parameters in terms of number of filters, filter size, number of hidden layers, dropout ratios and other parameters [49]. For instance, in our analysis, we have examined different filter lengths as well as batch normalization and dropout methods to determine the reported CNN model. Another challenge that is associated with deep learning techniques is the need for large data sets to be able to train the large number of hyperparameters that typically exist in deep learning models. However, with the rapid developments in the deep learning field, these challenges are expected to be resolved.

The generalization ability of the proposed model has been examined using two different experiments: training and testing the model using two non-overlapping sets of checkerboard patterns, in addition to training the model using checkerboard patterns and testing it using geometrical shapes patterns. The demonstrated ability of the model to generalize when applied to unseen patterns indicates its potential utility in the design of thalamic visual prosthesis. The proposed encoder could be used as an initial stage in visual prostheses to predict the activity of LGN neurons in response to a specific visual stimulus. The predicted firings can be then provided to a decoding stage that generates a corresponding electrical stimulus. The obtained electric stimulus would be used to artificially evoke neural firing similar to the activity that occurs under normal viewing conditions, thus resulting in a more accurate visual sensation [9]. In addition, the proposed model is not specific to a certain animal model. We aimed in this work to demonstrate, as a proof-of-concept using rat LGN data, the utility of the proposed CNN model, and more generally, deep learning, in modeling visual encoding. The choice of rats in this study is consistent with other studies that examined rat LGN firing properties to get insights on the visual encoding in the LGN in addition to visual prosthesis studies that relied on rats as target species [50–53]. However, this model could also be employed and extended to model visual encoding in other species. Moreover, the proposed encoding model is not limited to thalamic LGN only. It could be examined with other visual regions as in retinal and cortical visual encoding to improve the overall performance of other visual prostheses. Finally, extending this model to study the visual encoding along the visual pathway could provide insights into the intricate processing of the visual system.

## Conclusion

This paper introduced CNN-V-FH; a convolutional neural network visual encoding model of rat LGN that utilizes both the visual stimulus information and neuronal firing history. The model was used to predict rat LGN neural firing in response to different visual stimulation patterns achieving a mean correlation of 0.7 between the actual recorded and predicted firing rates. Our results showed the robustness of the proposed CNN strategy to different spatiotemporal properties of the recorded neurons. No significant difference was observed comparing the performance of the model for ON versus OFF neurons. Similarly, no significant difference was observed comparing the performance of the model for transient versus sustained firing neurons. Moreover, our results demonstrated the efficacy of the proposed model in comparison to other models. Specifically, for a firing rate window of 50 ms, the CNN-V-FH model achieved a mean enhancement in the performance of 68.1%, 88.5% and 98.8% of the neurons compared to the CNN-V, CNN-FH and GLM models, respectively. In addition, for the more precise firing rate window of 10 ms, the CNN-V-FH model achieved a mean enhancement in the performance of 79.4%, 82.1% and 93.8% of the neurons compared to the CNN-V, CNN-FH and GLM models, respectively. The model could provide insights about the LGN and how it encodes the visual information. It could also be used to guide the engineering of futuristic thalamic visual prostheses.

## Supplementary Information

**Additional file 1: Figure S1.** Samples of visual stimulation patterns presented in the experiments. (**a**) Single-pixel (4×8) patterns. (**b**) Checkerboard (4×8) patterns. (**c**) Shapes (900 × 1600) patterns. (**d**) Temporal properties of the presented stimulus pattern. **Figure S2.** Receptive Field of Recorded Neurons. (**a**) Normalized receptive fields of the same neurons given in Fig. 2 corresponding to the 4 × 8 screen obtained using spike-triggered average (STA). (**b**) Overall normalized receptive field for all neurons in all subjects, computed by averaging the receptive field matrices of all the recorded neurons in the single pixel and checkerboard experiments. The figure demonstrates that all pixels in the presented 4 × 8 are represented in the receptive fields of the recorded neurons with slight preference to pixels in the bottom center. **Figure S3.** Trial-to-trial variability expressed as the standard deviation (STD) of the actual and estimated firing rates across trials for each neuron. Each point represents one neuron for a specific stimulation pattern for (**a**) *w* = 10ms (*r* ^2^ = 0.602, *P* < 1e−^33^) and (**b**) *w* = 50ms (*r *^2^ = 0.671, *P* < 1e−^33^)**.**

## Data Availability

The data sets used and/or analyzed during the current study are available from the corresponding author on reasonable request.
