# Supplementary material for: A deep convolutional visual encoding model of neuronal responses in the LGN
Source: Brain Inform. 2021 Jun 15;8(1):11. doi: 10.1186/s40708-021-00132-6 (PMC8206408; doi:10.1186/s40708-021-00132-6)
Supplement: Supplementary file 1 — Additional file 1: Figure S1. Samples of visual stimulation patterns presented in the experiments. (a) Single-pixel (4×8) patterns. (b) Checkerboard (4×8) patterns. (c) Shapes (900 × 1600) patterns. (d) Temporal properties of the presented stimulus pattern. Figure S2. Receptive Field of Recorded Neurons. (a) Normalized receptive fields of the same neurons given in Fig. 2 corresponding to the 4 × 8 screen obtained using spike-triggered average (STA). (b) Overall normalized receptive field for all neurons in all subjects, computed by averaging the receptive field matrices of all the recorded neurons in the single pixel and checkerboard experiments. The figure demonstrates that all pixels in the presented 4 × 8 are represented in the receptive fields of the recorded neurons with slight preference to pixels in the bottom center. Figure S3. Trial-to-trial variability expressed as the standard deviation (STD) of the actual and estimated firing rates across trials for each neuron. Each point represents one neuron for a specific stimulation pattern for (a) w = 10ms (r 2 = 0.602, P < 1e−33) and (b) w = 50ms (r 2 = 0.671, P < 1e−33). [file 40708_2021_132_MOESM1_ESM.docx]

**Supplementary Methods**

A separate regularized GLM for each neuron was created using the open-source Python package pyglmnet [[1](#_ENREF_1)]. Each GLM predicts a single neuron firing rate based on the presented visual stimulus spatiotemporal representation as well as the firing history of all neurons (i.e. same features presented to our deep learning model). Each GLM is trained by optimizing the parameters ($\beta_{i}, \beta_{in}^{h}$ and $\beta_{ip}^{v}$) based on minimizing a penalized negative log-likelihood function of the training dataset [[1](#_ENREF_1)]. Given that GLM training requires significant computational resources, we used the neuroscience gateway servers to train and test the GLM for fine resolution firing rate window *w* = 10ms [[2](#_ENREF_2)].

The GLM training approach we used in our study minimizes a penalized negative log-likelihood function of the training dataset to fit feature matrix **X** with output vector **y***_i_*. The output vector **y***_i_* is expressed as

| $\boldsymbol{y}_{i}={[S_{i}\left( 1 \right),S_{i}\left( 2 \right),\ldots,S_{i}\left( K \right)]}^{T}$ | (1) |
| --- | --- |

where each value in the output vector $S_{i}\left( k \right)$ represents the firing rate of neuron *i* at time bin *k*, where *k* varies from 1 to the total number of bins *K*. Each row in **X** represents the features to be used to estimate the corresponding value in the output vector, where each row in the feature matrix $\mathbf{X}\left( k \right)$ is expressed as

| $\mathbf{X}\left( k \right) =[\mathbf{X}_{\boldsymbol{n}}\left( k \right),n=1\to N;\mathbf{X}_{\boldsymbol{p}}\left( k \right),p=1\to P]$ | (2) | |
| --- | --- | --- |
| $\mathbf{X}_{\boldsymbol{n}}\left( k \right)=[S_{n}\left( k-1 \right),S_{n}\left( k-2 \right),\ldots,S_{n}\left( k-L \right)]$ | (3) | |
| $\mathbf{X}_{\boldsymbol{p}}\left( k \right)=[I_{p}\left( k \right),I_{p}\left( k-1 \right),\ldots,I_{p}\left( k-L \right)]$ | (4) |  |

where $\mathbf{X}\left( k \right)$ consists of the firing rates history $\mathbf{X}_{\boldsymbol{n}}\left( k \right)$ of all neurons followed by the visual stimulation $\mathbf{X}_{\boldsymbol{p}}\left( k \right)$for all pixels. The firing rates history $\mathbf{X}_{\boldsymbol{n}}\left( k \right)$ consists of the firing rate of neuron *n* from time bin *k* – 1 to *k* – *L+*1, while the visual stimulation $\mathbf{X}_{\boldsymbol{p}}\left( k \right)$ consists of the intensity of pixel *p* from time bin *k* to *k* – *L+*1.

**a b c d**

| **Single-Pixel**     | **Checkerboard**     | **Shapes**         | **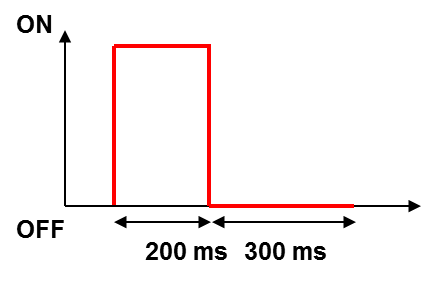 Trial Timing** |
| --- | --- | --- | --- |

**Fig. S1. Samples of visual stimulation patterns presented in the experiments.** (a) single-pixel (4×8) patterns. (b) checkerboard (4×8) patterns. (c) shapes (900 × 1600) patterns. (d) Temporal properties of the presented stimulus pattern.

**a**

**b**

**Fig. S2. Receptive Field of Recorded Neurons.** (a) Normalized receptive fields of the same neurons given in Fig. 2 corresponding to the 4 × 8 screen obtained using spike-triggered average (STA). (b) Overall normalized receptive field for all neurons in all subjects, computed by averaging the receptive field matrices of all the recorded neurons in the single-pixel and checkerboard experiments. The figure demonstrates that all pixels in the presented 4 × 8 are represented in the receptive fields of the recorded neurons with slight preference to pixels in the bottom center.

**a b**

**Fig. S3. Trial-to-trial variability expressed as the standard deviation (STD) of the actual and estimated firing rates across trials for each neuron.** Each point represents one neuron for a specific stimulation pattern for (a) *w* = 10ms (*r* ^2^ = 0.602, *P* < 1e^-33^) and (b) *w* = 50ms (*r*^2^ = 0.671, *P* < 1e^-33^).

**Supplementary Discussion**

Our analysis of the recorded single units revealed that the majority of the recorded rat LGN neurons possessed a transient firing pattern in response to the visual stimulus rather than a sustained firing pattern. This is consistent with other studies that reported an abundance of transient response in the LGN of rodents [[3](#_ENREF_3), [4](#_ENREF_4)]. One plausible interpretation is that Magnocellular LGN neurons in primates show more transient response than Parvocellular ones [[5](#_ENREF_5)]. The same is observed for Y-cells in cat LGN compared to X-cells [[6](#_ENREF_6)]. In addition, Magnocellular-like cells are believed to encode temporal information with low spatial resolution and no representation of color, whereas Parvocellular-like cells encode higher spatial resolution [[7](#_ENREF_7)]. Therefore, given those findings in addition to the low visual acuity of unpigmented rats and the mostly non-colored vision, the prevalence of transient response neurons (Magnocellular-like) could be explained.

The performance of the proposed model was examined with respect to multiple experimental and analysis factors. In terms of experimental factors, the performance of the model was not significantly variable across different stimulation patterns (i.e. single-pixel versus checkerboard) achieving correlation that exceeded 0.9 for some neurons. In the analysis, the model was able to predict the firing of LGN neurons at a fine resolution of 10ms with maximum correlation of 0.884. The diminished performance attained for some neurons could be explained given their low responsiveness to the presented stimuli as indicated by the responsiveness index analysis. Such low responsiveness could result from unadjusted stimulus luminance and/or contrast that are known to have a significant non-linear impact on the response of LGN neurons [[5](#_ENREF_5), [8](#_ENREF_8)]. It could also indicate neurons with receptive fields that are outside the visual field covered by the visual stimulation, or highly responsive, albeit distal, LGN neurons at the border of the recording electrode pickup vicinity [[9](#_ENREF_9)]. In our analysis, we sought not to discard those neurons from the analysis to examine the performance of the model under such non-optimal conditions.

**References**

1. Ramkumar P, Dyer E, Antalek M, Acuna D, Marques V. Pyglmnet 1.0.1.

2. Sivagnanam S, Majumdar A, Yoshimoto K, Astakhov V, Bandrowski A, Martone ME, et al., editors. Introducing the Neuroscience Gateway. IWSG; 2013.

3. Tang J, Jimenez SCA, Chakraborty S, Schultz SR. Visual receptive field properties of neurons in the mouse lateral geniculate nucleus. PloS one. 2016;11(1):e0146017.

4. Lennie P, Perry V. Spatial contrast sensitivity of cells in the lateral geniculate nucleus of the rat. The Journal of physiology. 1981;315(1):69-79.

5. Maunsell JH, Ghose GM, Assad JA, McADAMS CJ, Boudreau CE, Noerager BD. Visual response latencies of magnocellular and parvocellular LGN neurons in macaque monkeys. Visual neuroscience. 1999;16(1):1-14.

6. Derrington A, Fuchs A. Spatial and temporal properties of X and Y cells in the cat lateral geniculate nucleus. The Journal of physiology. 1979;293(1):347-64.

7. Denison RN, Vu AT, Yacoub E, Feinberg DA, Silver MA. Functional mapping of the magnocellular and parvocellular subdivisions of human LGN. Neuroimage. 2014;102:358-69.

8. Solomon SG, Peirce JW, Dhruv NT, Lennie P. Profound contrast adaptation early in the visual pathway. Neuron. 2004;42(1):155-62.

9. Buzsáki G. Large-scale recording of neuronal ensembles. Nature neuroscience. 2004;7(5):446.
